# Tailoring magnetism in silicon-doped zigzag graphene edges

**DOI:** 10.1038/s41598-022-16902-z

**Published:** 2022-07-29

**Authors:** Andoni Ugartemendia, Aran Garcia−Lekue, Elisa Jimenez−Izal

**Affiliations:** 1 Polimero eta Material Aurreratuak Fisika, Kimika eta Teknologia Saila, Kimika Fakultatea, Euskal Herriko Unibertsitatea (UPV/EHU), M. de Lardizabal Pasealekua 3, Donostia, Euskadi Spain; 2 Donostia International Physics Center (DIPC), M. de Lardizabal Pasealekua 3, Donostia, Euskadi Spain; 3 IKERBASQUE, Basque Foundation for Science, Bilbao, Euskadi Spain

**Keywords:** Chemistry, Materials science

## Abstract

Recently, the edges of single-layer graphene have been experimentally doped with silicon atoms by means of scanning transmission electron microscopy. In this work, density functional theory is applied to model and characterize a wide range of experimentally inspired silicon doped zigzag-type graphene edges. The thermodynamic stability is assessed and the electronic and magnetic properties of the most relevant edge configurations are unveiled. Importantly, we show that silicon doping of graphene edges can induce a reversion of the spin orientation on the adjacent carbon atoms, leading to novel magnetic properties with possible applications in the field of spintronics.

## Introduction

Spintronics has been regarded as a very promising field to design next generation information storage devices and logic gates^1,2^. In recent years, graphene materials have emerged as ideal elements in spintronic devices due to the possibility of combining spin localization and diffusion^3,4^. Contrary to precious transition metals which are in many cases scarce, toxic or whose mining presents socio-economic issues, carbon is one of the most abundant element on earth. In this sense, graphene could allow the large-scale production of these devices by means of a *greener* and more sustainable chemistry. Among the different graphene-based materials, structures with zigzag (ZZ) terminating edges such as nanoribbons (NR) and nanoholes have attracted widespread attention^5–7^, since a pure spin polarized current can be achieved along the edge. That is, the π electrons of edge carbon atoms present very flat bands near the Fermi energy and form localized states which align in a ferromagnetic (FM) fashion. Recently, it has been suggested that edge doping can be used to induce changes in the local magnetic moments of the carbon atoms^8–12^. B, N and Si are particularly interesting as they are similar to carbon in size and electronic structure.

Such edge decoration would require an atom-by-atom manipulation which until some decades ago would not be but a dream. However, the advent of aberration-corrected scanning transmission electron microscopy (AC-STEM) has laid a path towards this realization. Firstly, AC-STEM was viewed simply as an atomic scale imaging tool.^13^ Nonetheless, improvements in the electron beam (e-beam) sensitivity revealed that the e-beam could be used to move individual atoms and build nanostructures with atomic precision^14–18^. In this vein, AC-STEM has been used to direct Si atoms and small clusters in a graphene lattice, as well as along the graphene edges^19–24^. For instance, Chen et al*.* in ref^23^ created a nanohole in a single-layer graphene with a focused e-beam. Subsequently, residual silicon atoms from graphene growth migrated and attached to the edges. These experiments were performed at high temperatures (800 °C) with the aim of removing the chemical etching. Similarly, Ziatdinov et al*.* in ref 24 used a beam irradiation to open a hole in graphene and induce reactions of silicon atoms on the edge of the nanohole. Then, a combination of deep learning networks and a Gaussian mixture model was used to estimate some of the geometries of the doped edges.

Although Si-decorated graphene edges have been achieved experimentally via AC-STEM, an atomic level understanding of the electronic and magnetic properties of these systems is still lacking. During the experimental preparation of silicon decorated graphene edges several configurations were observed^21,23,24^. Given that ZZ edges are structurally richer, thermodynamically more stable and prone to show localized magnetic moments, in this work we focus on the effect of Si-doping the ZZ edges of graphene.

With this aim, a wide range of zigzag graphene nanoribbons (ZGNR) with Si atoms at or near the edges are considered. In particular, we study all the possible structures with one or two substitutional Si dopants at the edge or near-edge doping sites (see Figure S1) in a perfect ZGNR and a ZGNR containing one C vacancy at the edge. Note that in the latter a pentagon is formed as a result of this vacancy. The edges are left unsaturated so as to mimic the high mobility of H atoms in AC-STEM experimental conditions^23^. Density functional theory (DFT) is then employed to determine the exact geometry and thermodynamic stability of this set of experimentally inspired configurations, and to unravel how the electronic and magnetic properties are changed upon such doping. In particular, we concentrate on those Si-doped edge conformations in which a local inversion of the edge spin-polarization is induced.

## Results and discussion

**Figure 1:**
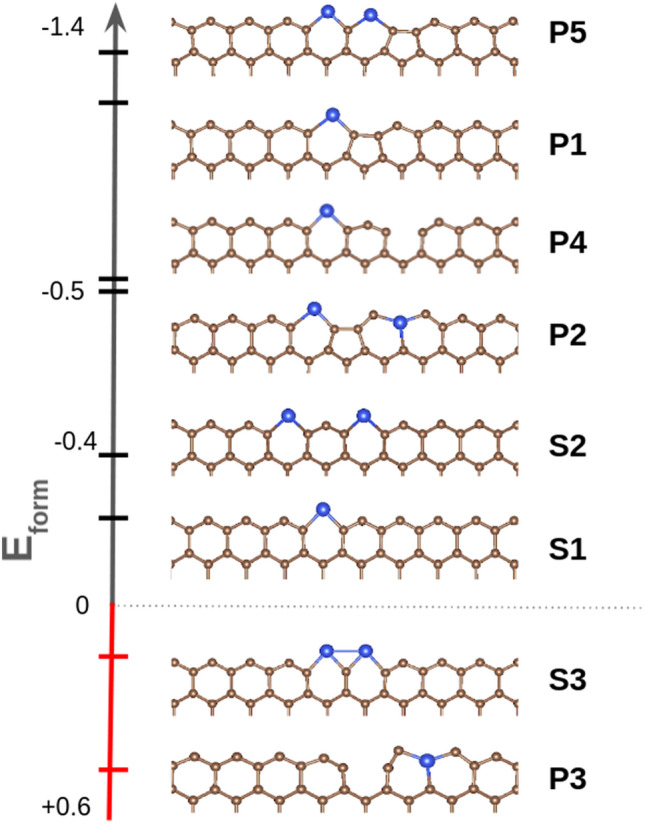
Representative Si-doped ZGNR structures along with their formation energies (*E*_form_) in eV. Carbon and silicon atoms are depicted in brown and blue, respectively.

First, the stability of Si edge doping in ZGNRs is studied. When one or two silicon atoms replace carbon atoms on the ZGNR, in the absence of vacancies (structures **S1**–**S8**, shown in Figure S3), most of the resulting structures are thermodynamically unstable, as revealed by their positive formation energies. There are only two exceptions, i.e. structures **S1** and **S2**, shown in Fig. 1. In **S1** one Si atom replaces one C atom at the sublattice A. This geometry, which has been experimentally observed by Chen et al.^23^ has a formation energy of − 0.29 eV. At the edge, the C–C bond length is 1.37 Å and the Si–C bond length is 1.87 Å, in perfect agreement with the experimental data^23^. This structure is planar, suggesting a sp^2^ hybridization on Si. **S2** is similar to **S1**, where two Si atoms at sublattice A are separated by an undoped hexagon. Its computed formation energy is slightly larger than that of **S1** (− 0.29 vs. 0.39 eV). Note that having two consecutive Si atoms at the sublattice A is thermodynamically unstable, as in **S3** shown in Fig. 1.

When a vacancy is created at the edge (structures **P1**–**P17**, shown in Fig. S4), there is more room to accommodate the dopant atoms and, as a result, the overall stability is significantly enhanced. A clear example can be found when comparing structures **S3** and **P5** and **P3**, shown in Fig. 1. In both structures two consecutive Si atoms are located on sublattice A. In **S3** the two Si atoms are squeezed in and the Si–Si bond length is 2.51 Å. The computed formation energy is + 0.13 eV. In **P5**, however, the Si atom next to the vacancy (the pentagon) is displaced towards such vacancy, moving away from the second Si atom. The resulting Si–Si distance is elongated up to 2.81 Å, and the formation energy is increased up to -1.33 eV. Likewise, the most thermodynamically stable structure is **P3**, where two Si atoms at the sublattice A are separated by a pentagon. The vacancy between them yields enough space for the dopant atoms to fit loosely, providing a large stability. Overall, the computed formation energies reveal the preference for Si atoms to be at the sublattice A, namely, at the edge row, rather than sublattice B, due to the larger size of Si as compared to C. In fact, thermodynamically unstable structures always have at least one Si atom at sublattice B (see for example **P3** in Fig. 1).

**Figure 2:**
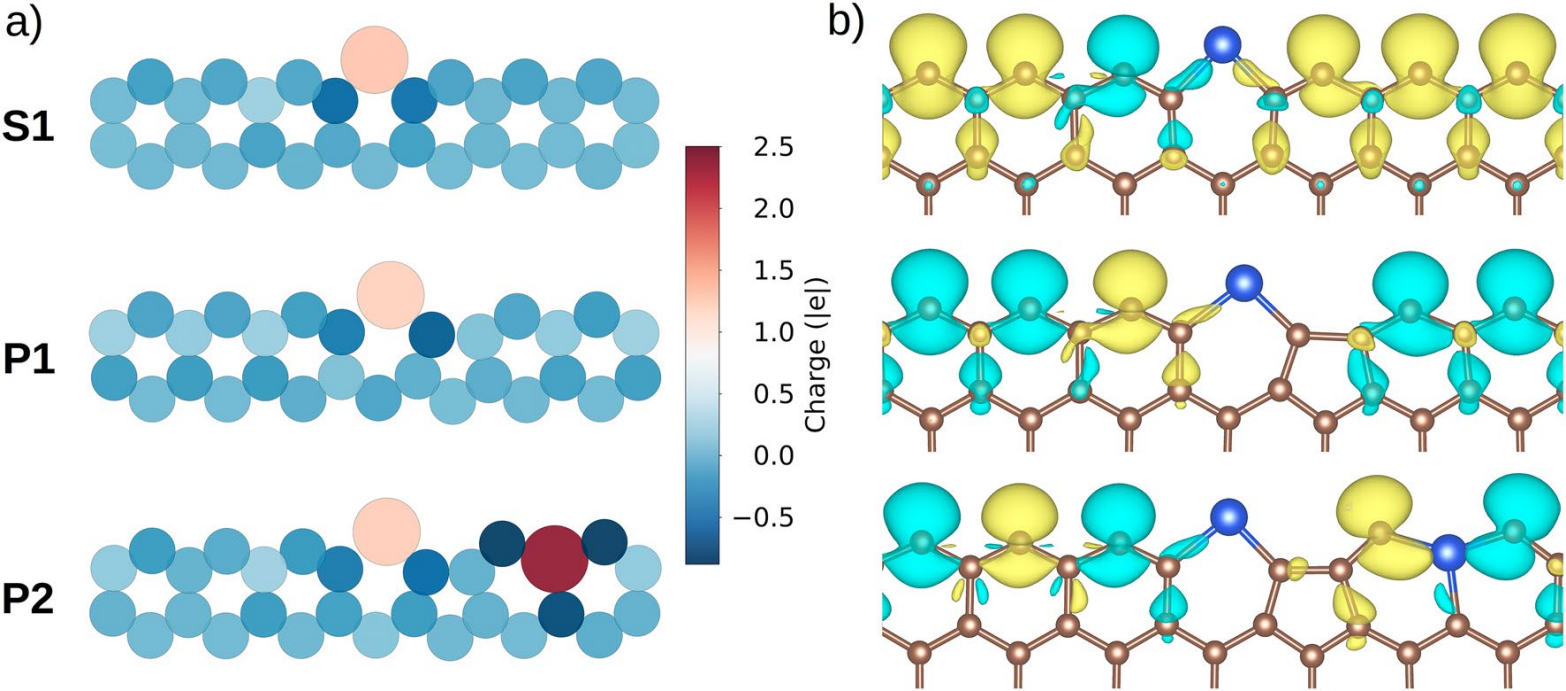
(**a**) Bader charges and (**b**) spin density on the top edge of selected **S1**, **P1** and **P2** ground state structures. The values for yellow (α-spin) and blue (β-spin) isosurfaces are 0.005 e/Å^3^. Carbon, and silicon atoms are depicted in brown and blue, respectively.

Due to the large number of structures modeled in this work, for the sake of clarity we have selected representative cases and studied their electronic and magnetic properties in-depth. Specifically, **S1** is chosen as representative of the experimentally detected structures, while **P1** and **P2** are selected due to their thermodynamic stability and interesting magnetic properties with varying silicon concentration (**P1**, E_form_ = − 1.26 eV, and **P2**, E_form_ = − 0.53 eV). First of all, the computed Bader charges depicted in Fig. 2a) show that there is a charge transfer of *ca.* 1.2 electrons from Si to C, leading to C atoms with partial negative charges on the edges. The reason for this charge transfer can be found in the electronegativity difference between both elements (2.5 for C vs 1.8 for Si). As shown in the computed charges of the **P2** structure, when Si is located in sublattice B, there are three Si–C bonds instead of just two, as in sublattice A, and the dopant exhibits a greater positive charge.

In order to study the changes induced by Si on the magnetic properties of graphene edges, first the undoped and unpassivated ZGNR are characterized. The spin density (Δ*ρ* = *ρ*↑ − *ρ*↓) is shown in Figure S5 of the Supplementary Information. As previously reported,^25,26^ the unpaired electrons within the edges are always coupled ferromagnetically (FM), while the antiferromagnetic (AFM) coupling between the edges is slightly preferred over the FM (by 60 meV, Figure S5). The magnetic moment on each C atom in sublattice A is 0.65 μ_B_. The spin density of the top edge of the selected **S1**, **P1** and **P2** structures is plotted in Fig. 2b) (the reader is referred to Figure S6 for the spin density of the whole ZGNR). The magnetic moment of the system mainly comes from the carbon atoms on the edges; all the spin density is located on these atoms. To study how the decorated edges behave magnetically, we considered the FM and AFM couplings between the edges. Regarding **S1**, in the most stable configuration the magnetic coupling between the unpaired electrons on opposite edges is AFM (Figure S6), as it is in the undoped ZGNR. Surprisingly, as shown in Fig. 2b), it is found that Si induces a flip of the electron spin on one of the C atoms located in the next sublattice A position. The magnetic moment carried by that C atom is 0.39 μ_B_ and the magnetic moment on the C atom on the other side of Si is 0.48 μ_B_. The rest of the C atoms in sublattice A, further away from Si, carry 0.65 μ_B_, similar to the undoped ZGNR, suggesting that the effect of Si on the spin density is only local.

**P1** structure is similar to **S1**, but with a carbon vacancy that leads to the formation of a pentagon next to Si. In this case, a spin flip on the C atom next to Si is also observed. The magnetic moment on this C atom is 0.43 μ_B_. In the most stable state the coupling between the edges is AFM (see Figure S6). Other configurations with different magnetic couplings are shown in Figure S6. Finally, the structure **P2** has the particularity of holding two Si atoms and, as a result, the obtained magnetic states are more diverse (see Figure S6). In the ground state the magnetic coupling between the edges is FM; and in this case the unpaired electrons of two C atoms on sublattice A are flipped. The magnetic moment on the C atoms range from 0.43 to 0.52 μ_B_.

Since magnetism can be sensitive to the employed model, we also explore the effect of different parameters on the magnetic properties of the selected structures (details in the Supplementary Information). First, to prove that the electron spin flipping is influenced only by Si doping and is independent from the interaction between opposite edges, the **S1**, **P1** and **P2** structures are characterized with reduced and enlarged ribbon widths, as shown in Figure S7. Specifically, ZGNR with *N* = 5 and *N* = 9 (84 and 140 atoms per unit cell, respectively) are characterized. In the case of **S1** and **P1** the coupling between edges is AFM and, as observed in the reference model, a spin flip occurs on the adjacent C atom, regardless of the ribbon width. In **P2** the coupling between opposite edges is FM, in agreement with the reference model. Shortening or widening the ZGNR leads to unchanged results, with the unpaired electrons on two C atoms flipped.

Second, the **S1**, **P1** and **P2** asymmetric structures are built, with one of the edges undoped and saturated with hydrogen. As can be seen in Figure S7, the hydrogenated C atoms exhibit a smaller magnetic moment (0.14 μ_B_). But, on the Si decorated edge, the magnetic moment and spin orientation of the C atoms is the same as in the reference model. Therefore, the novel and unexpected magnetic properties found in the Si doped graphene edges are only caused by such edge decoration.

Previous works analyze the effect of various dopants on the magnetic properties of similar graphene-based systems^27–29^, although not on Si–doped ZGNR edges. In this work, with the goal of elucidating the origin behind the predicted magnetic behavior of the doped edges, structures analogous to **S1**, **P1** and **P2** are built replacing the silicon atoms by carbon and keeping the geometries fixed (see Figure S8). The spin orientation of these structures is equal to the Si doped ZGNR, suggesting that the effect that Si exerts on the magnetism of the edges has a geometric origin, arising from the distortion caused by the larger size of Si compared to C. Structures **P1** and **P2** are geometrically asymmetric due to the C vacancy. In the same line, a closer inspection of **S1** indicates that there is an asymmetry in the area where the dopant atom is located. As depicted in Figure S9, Si is slightly tilted toward the C whose spin is flipped, resulting in a shorter C–Si distance (2.59 Å vs. 2.62 Å). Moreover, this geometric asymmetry causes a charge imbalance, leading to a larger dipole moment between the carbon atom that exhibits the electronic spin flip and the adjacent carbon atom. The breaking of the symmetry stabilizes one of the magnetic states (the one where one of the adjacent C to Si exhibits the spin flip), in a way that resembles the Jahn–Teller effect^30–33^. Besides, it is interesting to compare substitutional doping with adatom doping. In the latter, the carbon rings at the edge are hardly distorted, being this distortion not sufficient to stabilize the magnetic state with a spin flip (Δ*E* = 0.23 eV) (see Figure S10).

**Figure 3:**
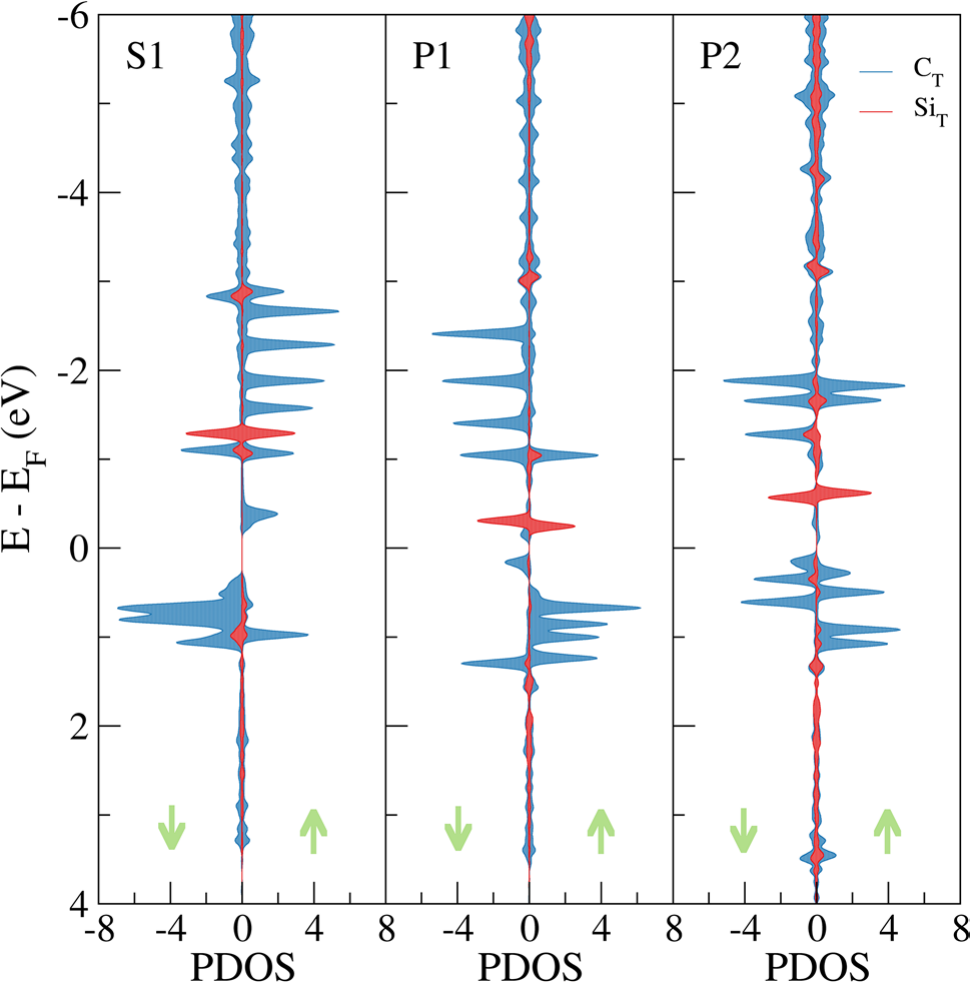
Projected density of states (PDOS) of the **S1**, **P1** and **P2** ground state structures. Positive and negative values correspond to α and β channels, respectively. Only the density of the top edge atoms is shown. The Fermi energy (*E*_F_) is set to zero in all cases.

The projected density of states (PDOS) of **S1**, **P1** and **P2** ground states are illustrated in Fig. 3. The PDOS of the other metastable magnetic states can be found in Figures S11–S18. The total DOS is projected onto the C (blue) and Si (red) atoms on the top edge. As can be observed, these structures give rise to highly localized and spin polarized states near the Fermi energy (E_F_). Such states correspond to the flat bands of the C edge atoms (six atoms in **S1** and five atoms in **P1** and **P2**). Si atoms in **S1** and **P1** also show edge states below the Fermi level. For, **P2**, which has Si atoms in both sublattices A and B, a closer inspection suggests that only the Si atoms in sublattice A form localized edge states while the Si states in sublattice B are extended over the region − 1.4 eV to − 0.8 eV. Besides, the structures show a strong hybridization between Si and C atoms, indicating the formation of Si–C covalent bonds which provide great stability to the system. In agreement with Bader charges, these bonds are slightly polarized since empty Si states can be observed above the E_F_.

It is well known that in nonmagnetic (NM) pure ZGNR, the valence and the conduction bands of the edge atoms converge at the E_F_ showing a half–filled peak. As can be observed in Fig. 3, the high DOS at the E_F_ causes a Stoner instability and consequently, the peak is splitted into filled and empty states leading to a FM ordering along the edge.^25^ The same splitting of the bands is observed in the Si doped ZGNR. In the top edge of **S1** there are five occupied α edge states in the range − 2.75 eV to − 1 eV. However, in agreement with the spin density analysis, the distortion caused by Si breaks the degeneracy of the FM coupling and the β (down) LUMO state is stabilized below the E_F_ (*ca.* − 1 eV), while the α (up) HOMO state is shifted above the E_F_, thus reverting the magnetic moment. Each of these states have their corresponding empty edge state in the opposite spin channel above the E_F_. In **P1** there are 4 occupied β edge states in the top edge, one less than in **S1** due to the vacancy. Likewise, the magnetic moment of the other edge atom is flipped and gives rise to an occupied α edge state at *ca.* − 1 eV. Unlike the previous cases, in **P2** two C atoms undergo a spin flip and thus, three edge states have β spin and the other two α spin. Summing up, substitutional Si doping modifies the stability of the edge states in both spin channels and consequently affects the magnetic moments. Spin density and PDOS analysis reveal that Si edge doping can be used to fine–tune the magnetic properties of ZGNRs.

**Figure 4:**
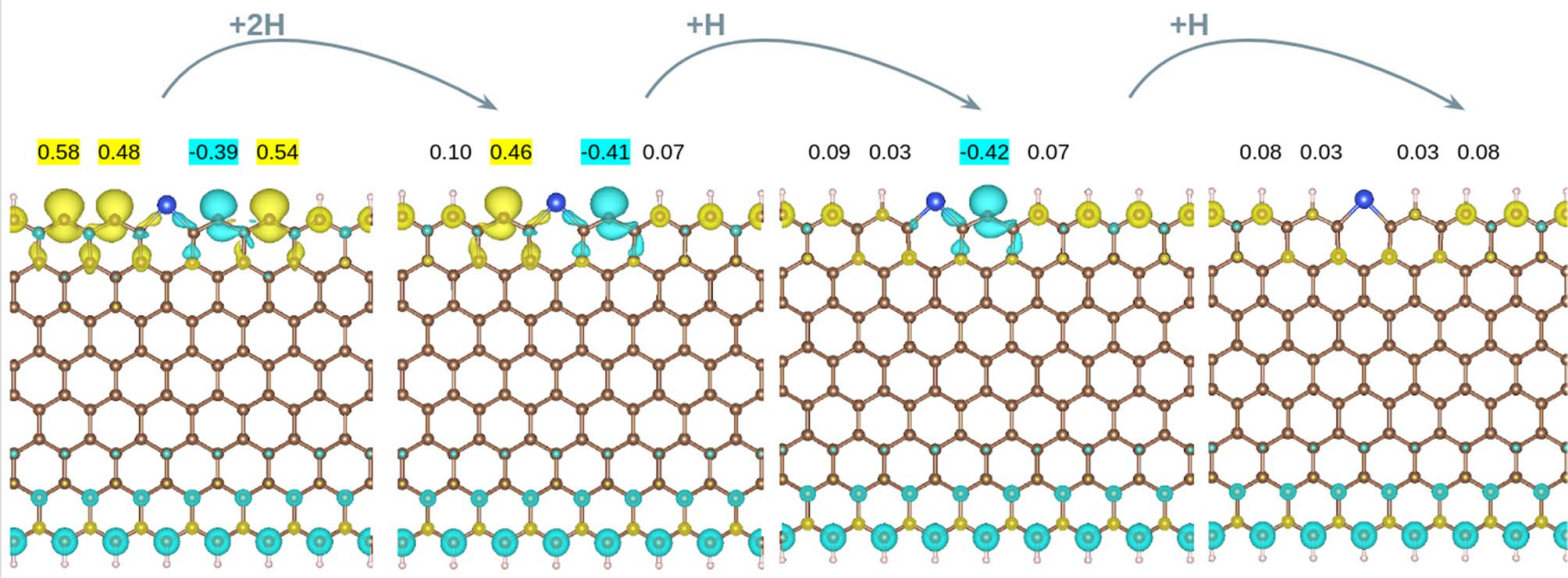
Spin density of **S1** with one edge undoped and passivated with H atoms and the opposite edge partially passivated with a varying number of H atoms. Isosurface value of 0.005 e/Å^3^. The values of the magnetic moments (in μ_B_) of the C edge atoms are also shown. The values for yellow (α-spin) and blue (β-spin) isosurfaces are 0.005 e/Å^3^. Carbon, silicon and hydrogen atoms are depicted in brown, blue and white, respectively.

It should be noted that unpassivated ZGNR edges are unstable and tend to either passivate in the presence of impurities or reconstruct into Stone–Wales defects^34,35^. For this reason, finally, we investigate how robust the predicted magnetic properties are and particularly, the spin reversion induced by Si, with respect to H passivation. We start from the **S1** asymmetric ZGNR; terminated by H on one edge while unterminated and Si-doped on the other. Then, H atoms are progressively added on the C atoms at the doped and unsaturated edge, as shown in Fig. 4. Interestingly, as long as the C atom next to Si is not passivated, the spin flip induced by the dopant and the magnetic moment on the C atom (*ca.* 0.4 μ_B_) remains unchanged. These results suggest that σ edge states are needed to observe such spin flip, probably because the magnetic moment arising from the π electrons is not large enough. Although these structures are expected to have a short lifetime due to their high chemical reactivity, our results introduce a novel procedure to tune the magnetic properties at Si–doped graphene ZZ edges. For instance, one could envision preserving the spin reversion by attaching a protecting group to the target C atom next to Si. Further work is intended in this line. Should such precise chemical engineering be experimentally attainable, a zoo of different magnetic structures would be at our disposal. The structures could be converted into one another by directing Si atoms along the edges. In this way, it would be possible to create localized magnetic moments of specific orientation, which would be advantageous in the design of spin–based logic gates.

In summary, density functional theory is applied to model and characterize silicon doped graphene edges, as those recently obtained experimentally. The thermodynamic stability is assessed and the electronic and magnetic properties of the most relevant edge configurations are unraveled. Importantly, we observe that the edge doping breaks the spin degeneracy of the edge states, leading to a variety of possible outcomes. In selected structures, an electronic spin symmetry breaking occurs upon doping, leading to a spin flip of one of the carbon atoms next to silicon. The role played by the ribbon width, the concentration of the dopant atoms along the ribbon’s edges, and the effect of doping just one or both edges symmetrically is elucidated. It is concluded that the observed spin flip effects are local, solely induced by Si and independent of the interaction between edges. Our results could pave the way towards a novel strategy to tune the spin–texture at Si–doped graphene ZZ edges, and contribute to the development of spintronic devices that exploit the edge decoration of graphene nanoribbons.

## Methods

Density functional theory (DFT) calculations were done with the PBE exchange-correlation functional^36,37^ and projector augmented wave (PAW) pseudopotentials,^38^ as implemented in the VASP code^39–41^. The planewave kinetic energy cutoff was set to 450 eV, and the convergence criteria for geometry (SCF) relaxation was set to 10^–5^ (10^–6^) eV. The DFT-D3 scheme was used to account for the dispersion interactions^42^. The convergence of different parameters was checked to ensure the reliability of our calculations, as explained in the Supplementary Information.

As shown in Figure S1, the carbon atoms at the center of the ribbon were fixed at positions of bulk (graphene), while the atoms on the edges and near-edge positions were allowed to relax. Si doping was tested on these positions. GNR was modeled using a unit cell with a length of 17.27Å in its growth direction *x*, containing a total of 112 atoms and a width corresponding to *N* = 7, as indicated in Fig. S1. The adjacent layers in y and z directions are separated by a vacuum space of at least 10 Å. Such distance was enlarged up to 30 Å and the results were unchanged, guaranteeing that 10 Å is enough to avoid spurious interactions between periodic images. In addition to planar geometries, silicon atoms were moved out of the plane to ensure that potential non-planar structures were also explored during the geometry optimization. The integration over the Brillouin zone was set using a 5 × 1 × 1 *k*-points grid. A more accurate Γ centered 25 × 1 × 1 k-point grid was used for the projected density of states (PDOS). A very fine energy grid was required in order to reproduce the localized states of the edge atoms. All the calculations were spin polarized.

The formation energies were calculated as

1 $$E_{{{\text{form}}}} = \left( {E_{{{\text{doped}}}} + n\mu_{{\text{C}}} - m\mu_{{{\text{Si}}}} - E_{{{\text{perfect}}}} } \right)/2,$$

where *E*_doped_ and *E*_perfect_ are the total energy of Si-doped and pristine GNR, *μ*_C_ and *μ*_Si_ are the chemical potentials of C and Si, calculated as the energy per C atom in graphene and the energy per Si atom is bulk silicon^43^, and *n* is the number of removed C atoms and *m* the number of Si atoms added to the doped NR. Note that the more negative the formation energy, the more thermodynamically stable the structure is.

## Supplementary Information

Supplementary Information.

## Data Availability

The data that support the findings of this study are available from the corresponding author upon reasonable request. The corresponding author, on behalf of all authors of the paper, is responsible for submitting a competing interests statement.
