# Supplementary material for: Tailoring magnetism in silicon-doped zigzag graphene edges
Source: Sci Rep. 2022 Jul 29;12:13032. doi: 10.1038/s41598-022-16902-z (PMC9338279; doi:10.1038/s41598-022-16902-z)
Supplement: Supplementary file 1 — Supplementary Information. [file 41598_2022_16902_MOESM1_ESM.pdf]

Supplementary Information

TAILORING MAGNETISM IN SILICON−DOPED ZIGZAG GRAPHENE EDGES

Andoni Ugartemendia,[1,2] Aran Garcia−Lekue,[2,3,*] and Elisa Jimenez−Izal[1,2,3,*]

[1]Polimero eta Material Aurreratuak: Fisika, Kimika eta Teknologia Saila, Kimika Fakultatea, Euskal Herriko Unibertsitatea (UPV/EHU), M. de Lardizabal Pasealekua 3, Donostia, Euskadi (Spain)
[2]Donostia International Physics Center (DIPC), M. de Lardizabal Pasealekua 3, Donostia, Euskadi (Spain)
[3]IKERBASQUE, Basque Foundation for Science, Bilbao, Euskadi (Spain)

E-mail: elisa.jimenez@ehu.es; wmbgalea@ehu.eus

Table of contents:

1. ZGNR model and convergence
2. Optimized structures along with their spin density
   2.1 Addition of one or two substitutional Si atoms
   2.1 Addition of one or two substitutional Si atoms in an edge with a carbon vacancy
3. Undoped and unpassivated ZGNR
4. Spin density of different magnetic states of the **S1**, **P1** and **P2** structures and the relative energy with respect to the magnetic ground state.
5. Effect of ribbon width and edge passivation
6. Spin density of the **S1**, **P1** and **P2** structures with silicon atoms replaced by carbon atoms and with frozen geometries
7. C-Si bond distances and the Bader charges on the **S1** structure.
8. Spin density on the most stable non–substitutional edge adsorption of Si on ZGNR
9. Projected density of states (PDOS)

1. ZGNR model and convergence

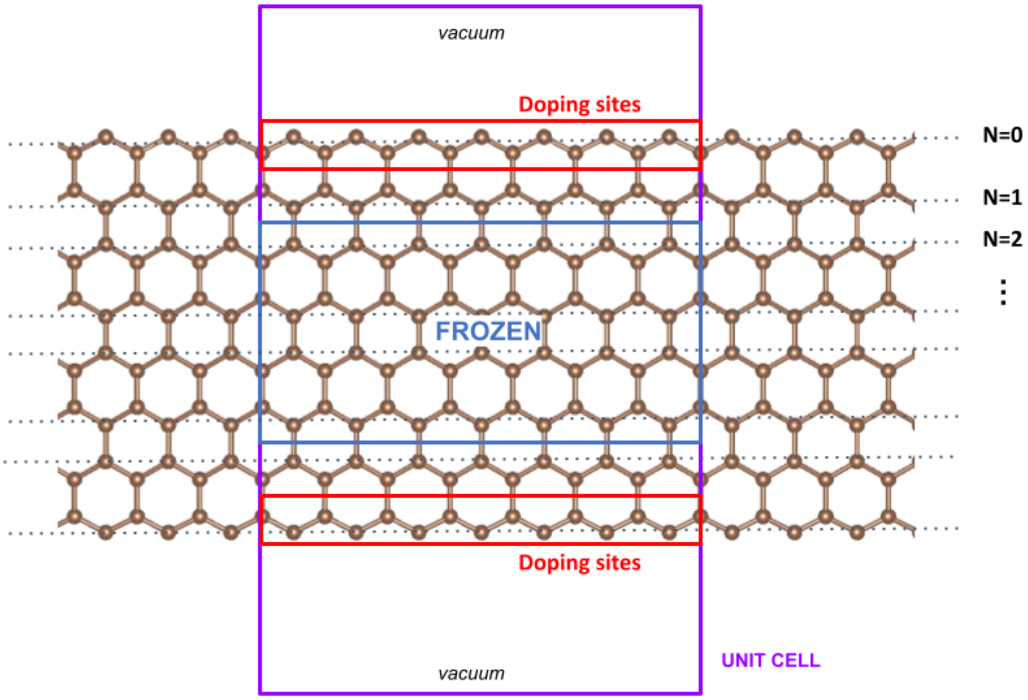

Figure S1. Schematic representation of the ZGNR model used in the present work.

In order to ensure the reliability of our calculations, different parameters were tested on undoped zigzag GNR.

A) *K*points number

As it can be observed in Fig. S2, the convergence of the number of *k*-points in the GNR growth direction, i. e., nx1x1 was tested. The convergence was achieved at 5x1x1, where the change in energy was smaller than 0.006 eV.

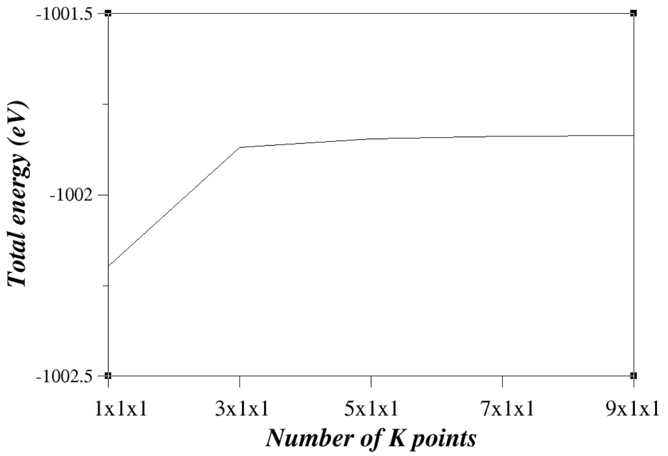

Figure S2. Convergence of the energy of ZGNR with the number of *k*-points.

B) Space between adjacent layers

The adjacent layers in GNR in *y* and *z* directions are separated by a vacuum space in order to avoid interactions between two layers. The dimension of this vacuum space was checked in the *y* direction, by using two different unit cells: 17.27 x **27** x 15 Å (spacing between cells of 11.32 Å) and 17.27 x **35** x 15 Å (spacing between cells of 19.44 Å). The results are shown in Table S1 for GNR with ferromagnetic (FM) and antiferromagnetic (AFM) coupling between edges. It is clear from these data that the convergence is already achieved in the smallest unit cell.

Unit cell	Energy difference between AFM and FM states (eV)
17.27 x **27** x 15 Å	0.06287097
17.27 x **35** x 15 Å	0.06288035

Table S1. Energy difference between AFM and FM states as a function of the vacuum space between cells and magnetic couplings between edges (FM stands for ferromagnetic coupling and AFM for antiferromagnetic coupling between edges).

2. Optimized structures along with their spin density

2.1 Addition of one or two substitutional Si atoms

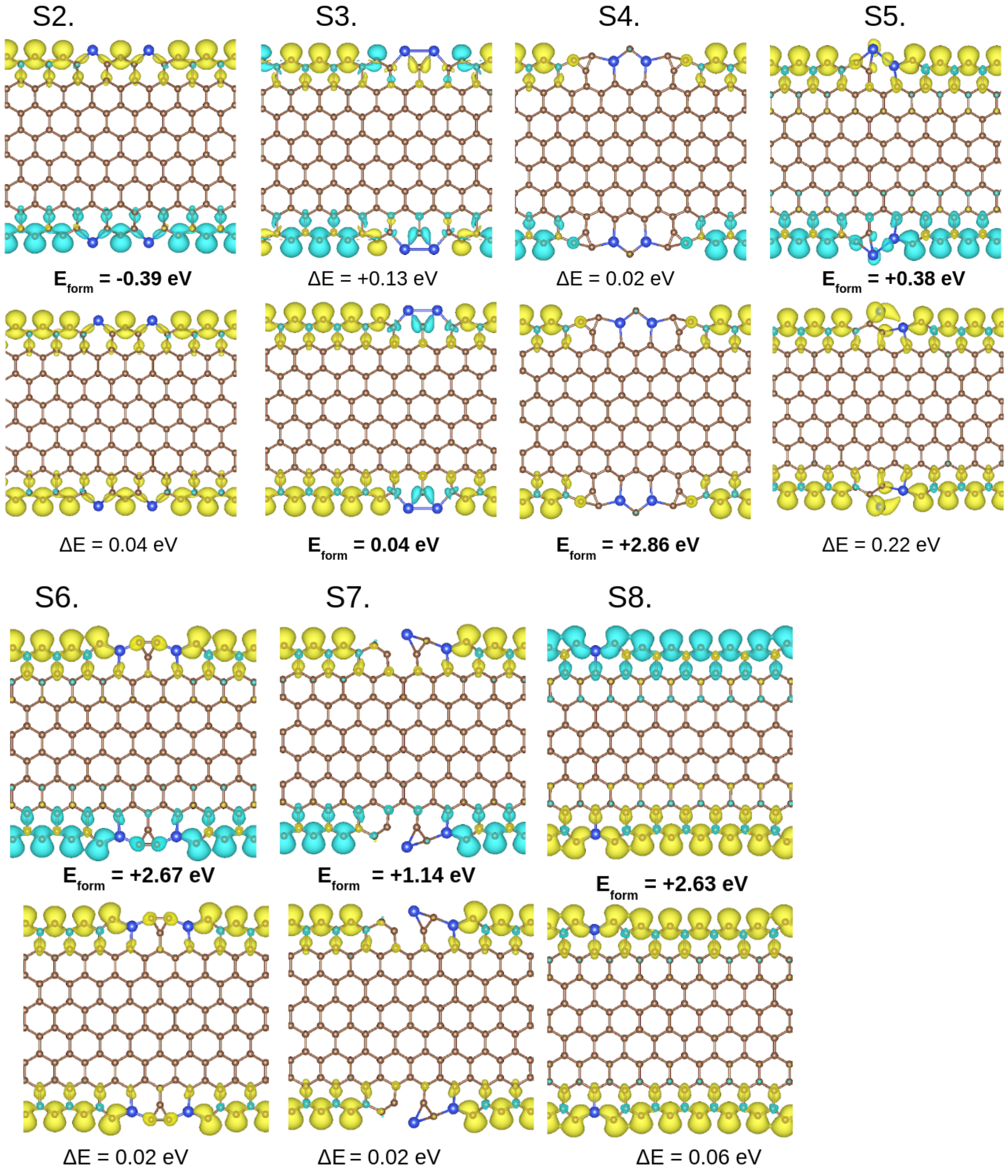

Figure S3. Spin density on structures **S2**–**S8**. For each case the state with AFM (top) and FM coupling between opposite edges are shown. The ground state is shown next to the formation energy of each structure (E_{form}), and the second most stable magnetic state is given next to the energy difference with respect to such ground state. The values for yellow (α–spin) and blue

(β–spin) isosurfaces are 0.005 e/Å^3. Carbon and silicon atoms are depicted in brown and blue respectively.

2.1 Addition of one or two substitutional Si atoms in an edge with a carbon vacancy

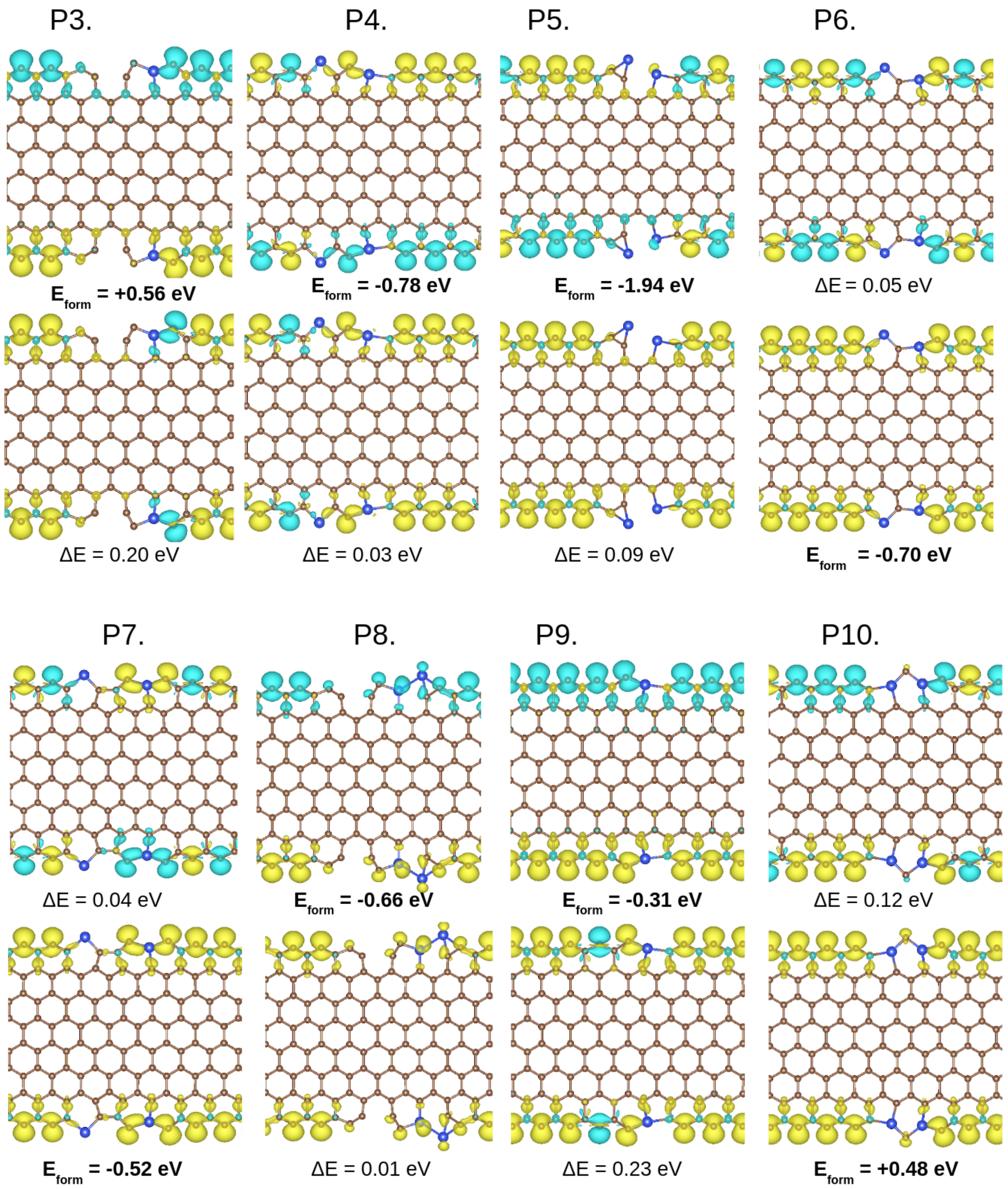

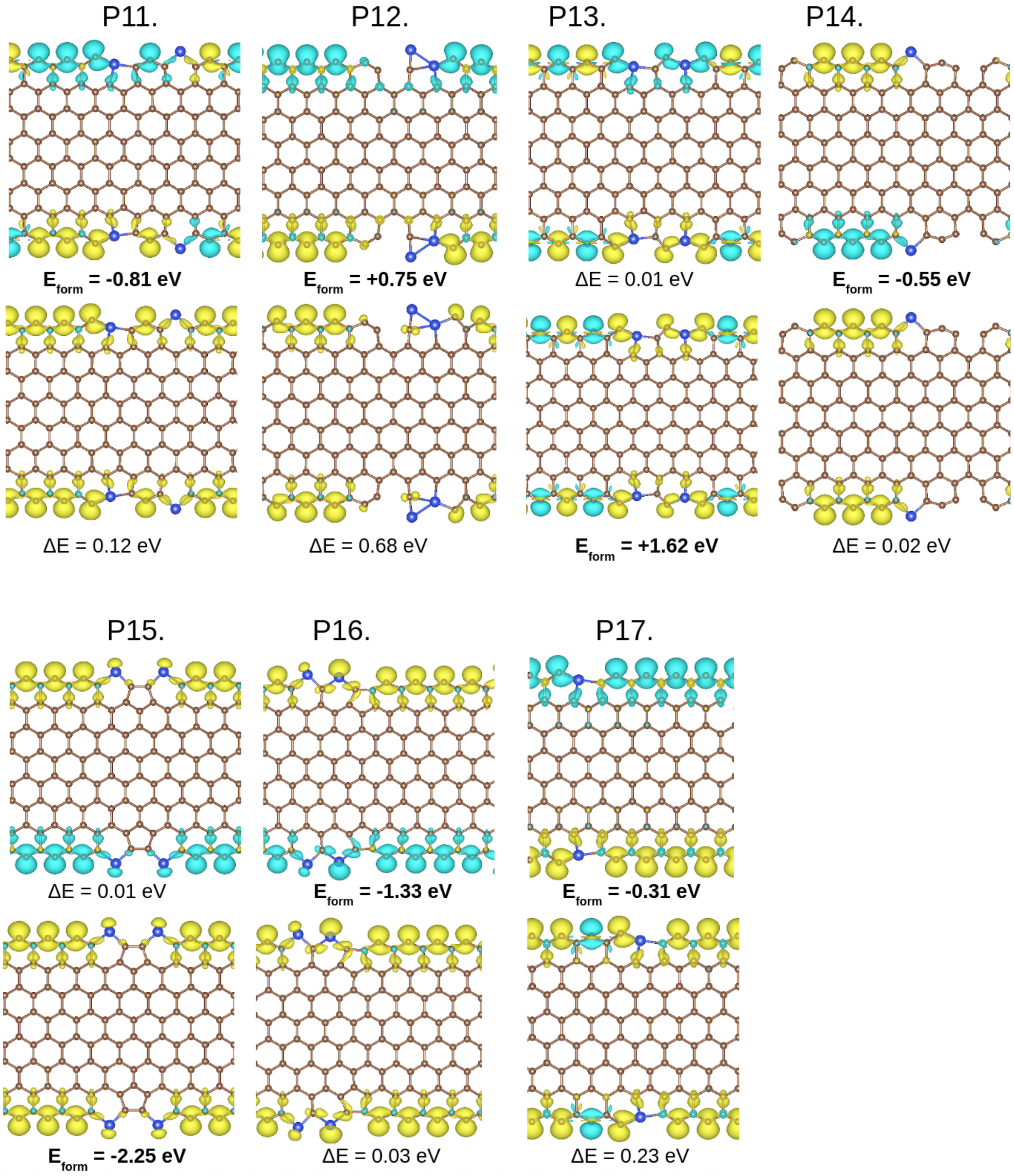

Figure S4. Spin density on structures **P3–P17**. For each case the state with AFM (top) and FM coupling between opposite edges are shown. The ground state is shown next to the formation energy of each structure (E_{form}), and the second most stable magnetic state is given next to the energy difference with respect to such ground state. The values for yellow (α–spin) and blue (β–spin) isosurfaces are 0.005 e/Å^3. Carbon and silicon atoms are depicted in brown and blue respectively.

3. Undoped and unpassivated ZGNR.

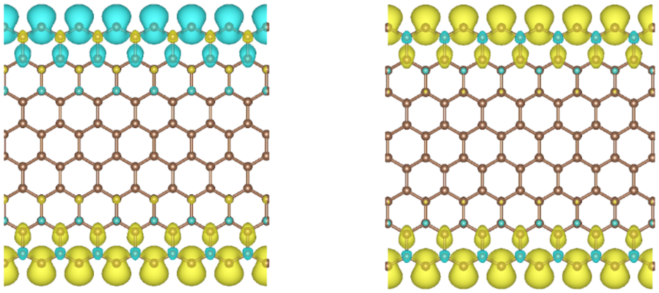

ΔE = 0.06 eV

Figure S5. Spin density on unpassivated and undoped ZGNR. On the left the ground state, i. e., AFM state, and on the right the FM state, that lies 0.06 higher in energy. The values for yellow (α–spin) and blue (β–spin) isosurfaces are 0.005 e/Å^{3}. Carbon and silicon atoms are depicted in brown and blue respectively.

4. Spin density of different magnetic states of the **S1**, **P1** and **P2** structures and the relative energy with respect to the magnetic ground state.

Regarding **S1**, in the most stable configuration the magnetic coupling between the unpaired electrons on opposite edges is AFM (Figure S6), as it is in the undoped ZGNR, where Si induces a flip of the electron spin on one of the C atoms located in the next sublattice A position. In addition, other magnetic states are characterized and shown in Figure S6. In the configuration closest in energy (only 30 meV higher) the spin flip effect induced by Si is found too. The difference is that the coupling between edges is FM. The magnetic state where all the carbon atoms are coupled ferromagnetically, with no spin flip, with either AFM and FM coupling between the edges is 90 and 130 meV higher in energy, respectively.

In **P1** structure a spin flip on the C atom next to Si is also observed. In the most stable state the coupling between the edges is AFM. The analogous configuration with a FM coupling between edges is 60 meV less stable (Figure S6). Unlike what is found in **S1**, in **P1** the magnetic state with FM coupling within each edge and AFM between edges is only 40 meV higher in energy. Thus the spin flip induced by Si in **S1** is slightly more robust.

In **P2** the ground state exhibits a FM magnetic coupling between the edges, where the unpaired electrons of two C atoms on sublattice A are flipped. Within 120 meV from the ground state other magnetic states are found where the electrons of two C atoms are flipped, as well as one state where the coupling within the edge and between the edges is FM (Figure S6).

S1

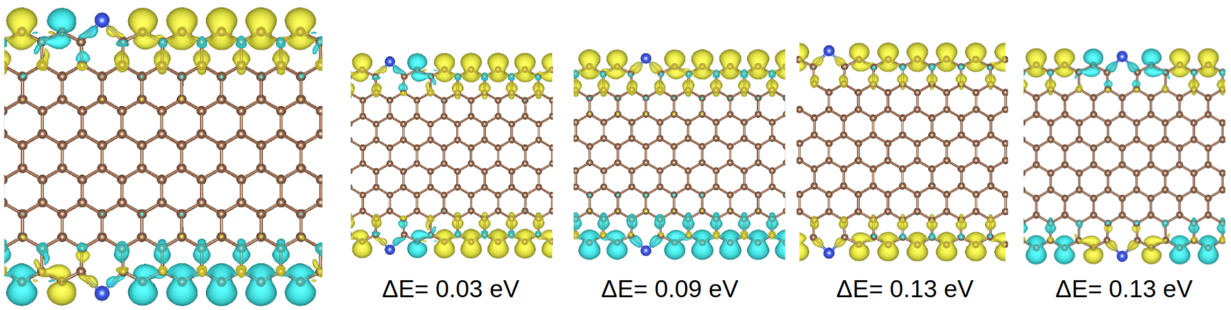

ΔE= 0.03 eV
ΔE= 0.09 eV
ΔE= 0.13 eV
ΔE= 0.13 eV

P1

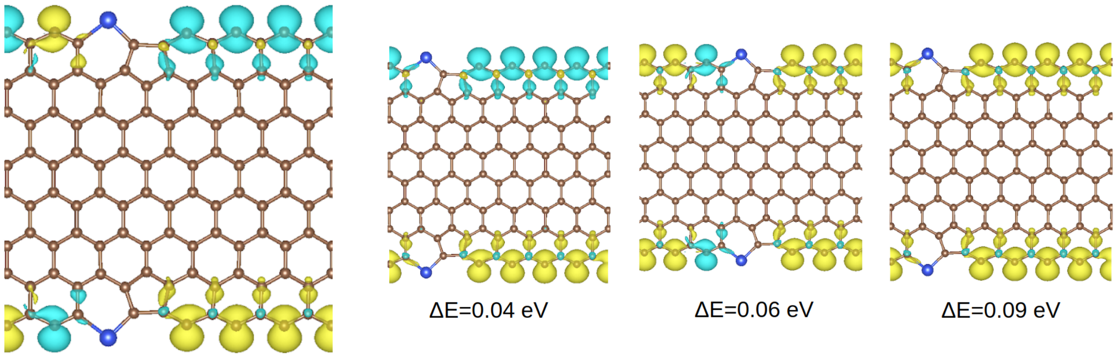

ΔE=0.04 eV
ΔE=0.06 eV
ΔE=0.09 eV

P2

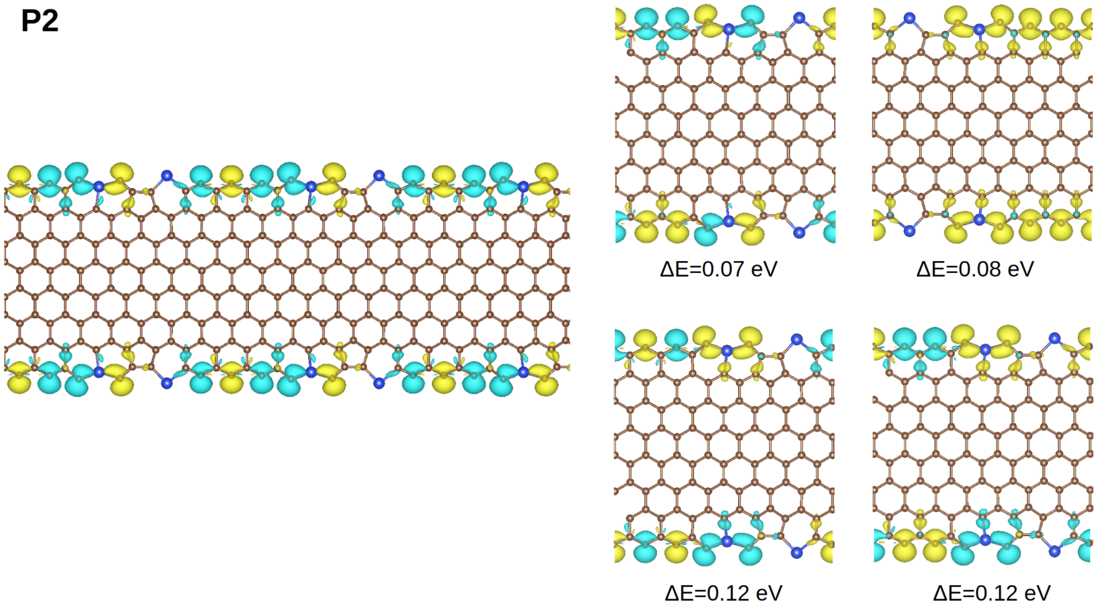

ΔE=0.07 eV
ΔE=0.08 eV
ΔE=0.12 eV
ΔE=0.12 eV

Figure S6. Spin density on selected **S1**, **P1** and **P2** cases. For each case the ground state is given as well as other found magnetic states and their energy difference with respect to the ground state. The values for yellow (α–spin) and blue (β–spin) isosurfaces are 0.005 e/Å^3. In the most stable state of **P1** three consecutive unit cells are shown for the sake of clarity. Carbon and silicon atoms are depicted in brown and blue, respectively.

5. Effect of ribbon width and edge passivation

S1

Reduced width

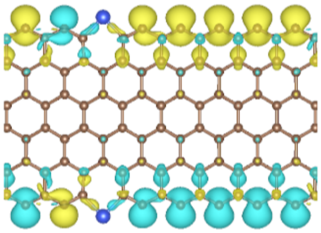

Enlarged width

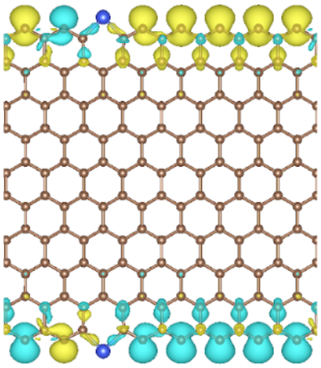

Single edge doped

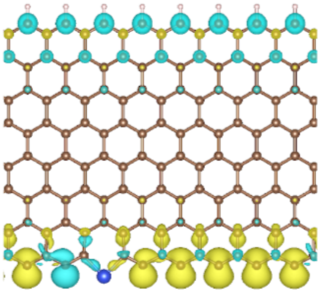

P1

Reduced width

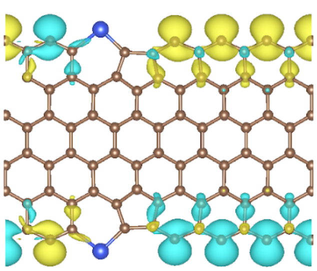

Enlarged width

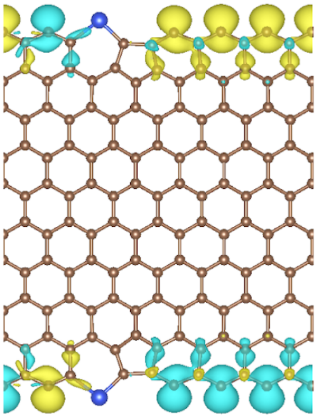

Single edge doped

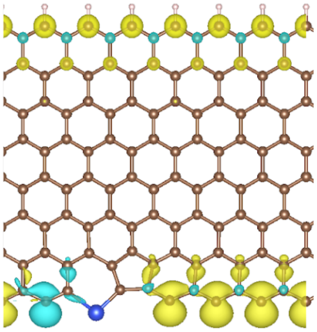

P2

Reduced width *Enlarged width* *Single edge doped*

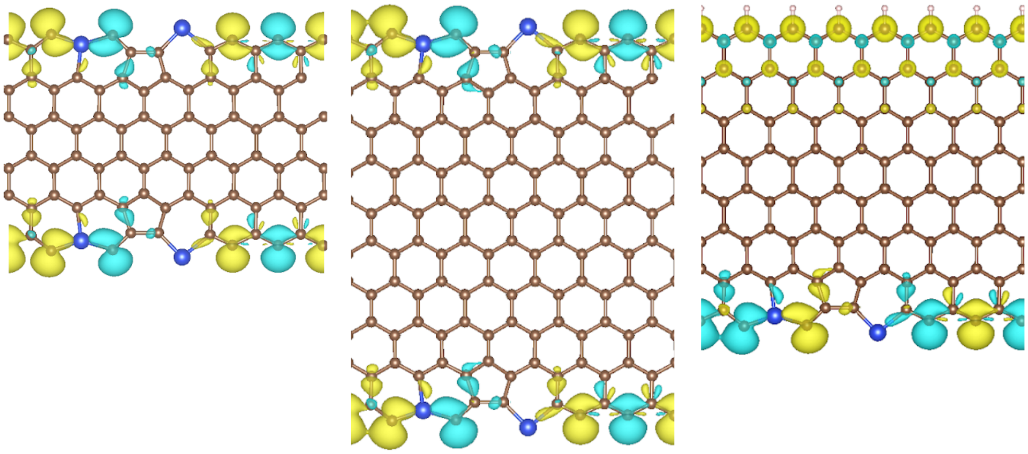

Figure S7. Spin density on selected **S1**, **P1** and **P2** ground state cases. The effect of ribbon width and edge passivation is compared. The values for yellow (α–spin) and blue (β–spin) isosurfaces are 0.005 e/Å^3. Carbon, silicon and hydrogen atoms are depicted in brown, blue and white, respectively.

6. Spin density of the **S1**, **P1** and **P2** structures with silicon atoms replaced by carbon atoms and with frozen geometries.

S1 **P1** **P2**

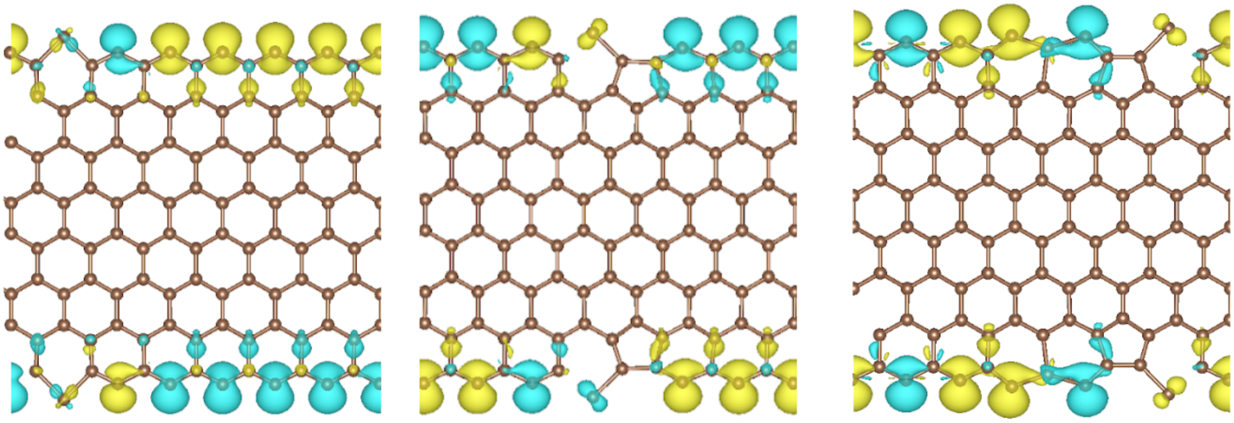

Figure S8. Spin density on selected **S1**, **P1** and **P2** cases where Si atoms have been substituted by C and the geometries have been kept fixed. The values for yellow (α–spin) and blue (β–spin) isosurfaces are 0.005 e/Å^3.

7. C–Si bond distances and the Bader charges on the **S1** structure.

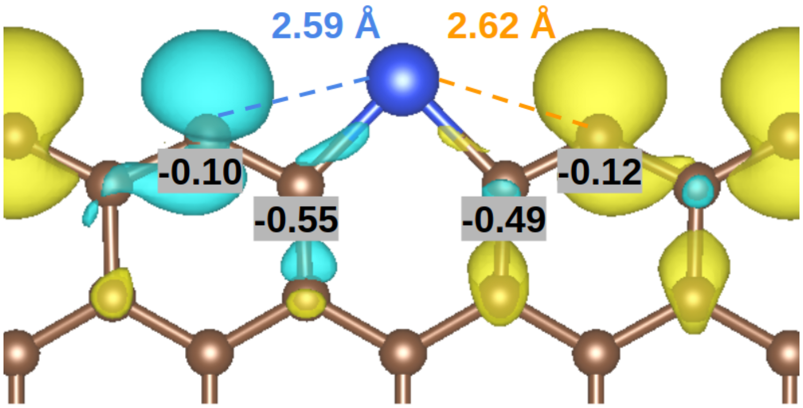

Figure S9. C-Si bond distances (Å), along with the bader charges (|e⁻|) around the Si atom on structure **S1**. The values for yellow (α–spin) and blue (β–spin) isosurfaces are 0.005 e/Å^3. Carbon and silicon atoms are depicted in brown and blue, respectively.

8. Spin density on the most stable non–substitutional edge adsorption of Si on ZGNR

The non–substitutional edge adsorption of Si was explored as a reference. The most stable structure of the Si adatom is shown in Figure S7 and consists of a Si atom bonded to two carbon atoms on the edge of the ZGNR. In this case, a spin-flip is observed only for a magnetic configuration lying 0.23 eV higher in energy than the ground state, which shows no inversion at all.

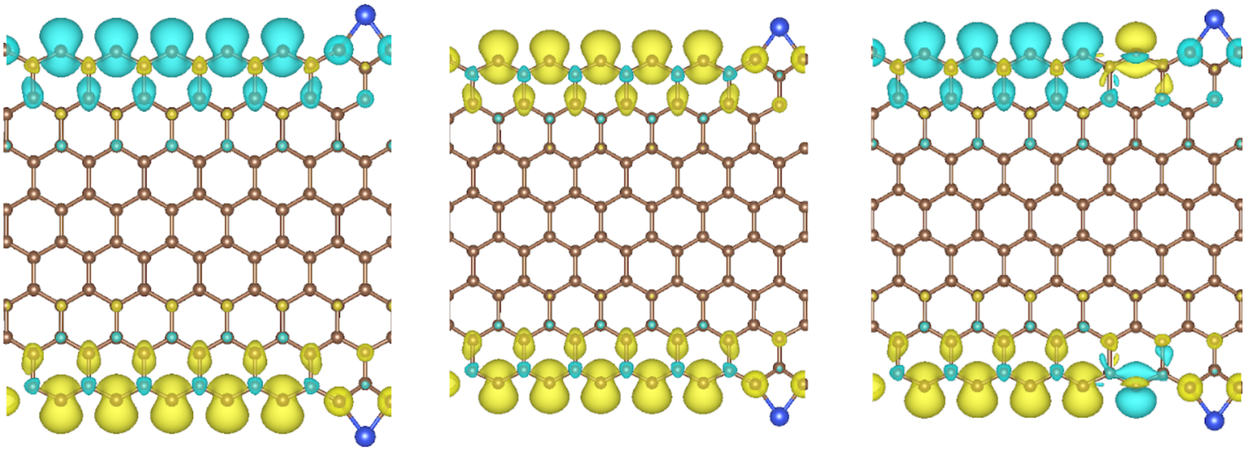

E_{form} = -1.37 eV　　ΔE = 0.05 eV　　ΔE = 0.23 eV

Figure S10. Spin density on the most stable non–substitutional edge adsorption of Si on ZGNR. The ground state is given, as well as other stable magnetic states and their energy difference with respect to the ground state. The values for yellow (α–spin) and blue (β–spin) isosurfaces are 0.005 e/Å^3. Carbon and silicon atoms are depicted in brown and blue, respectively.

9. Projected density of states (PDOS)

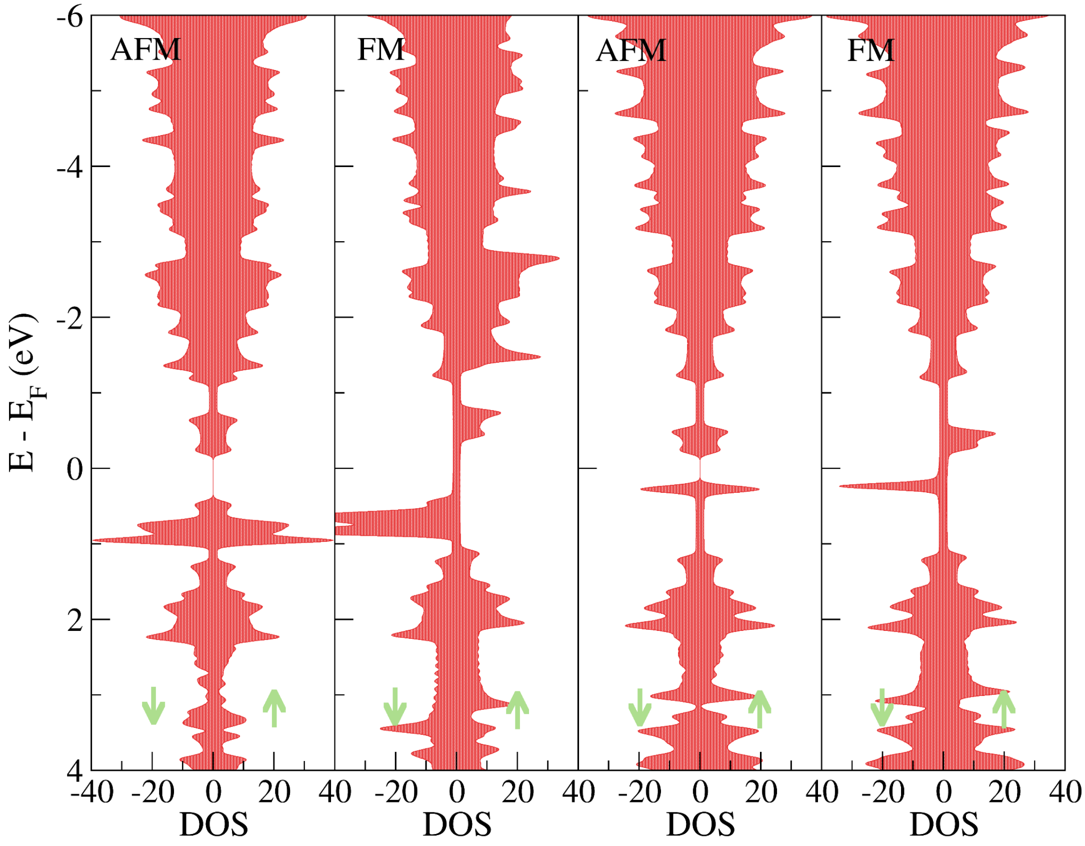

Figure S11. Total density of states (DOS) of undoped ZGNRs. The two leftmost PDOS correspond to unpassivated ZGNRs while the two rightmost ones correspond to passivated ZGNRs. AFM coupling is more stable in both cases. Positive and negative values correspond to α and β channels, respectively. The Fermi energy (E_F) is set to zero in all cases.

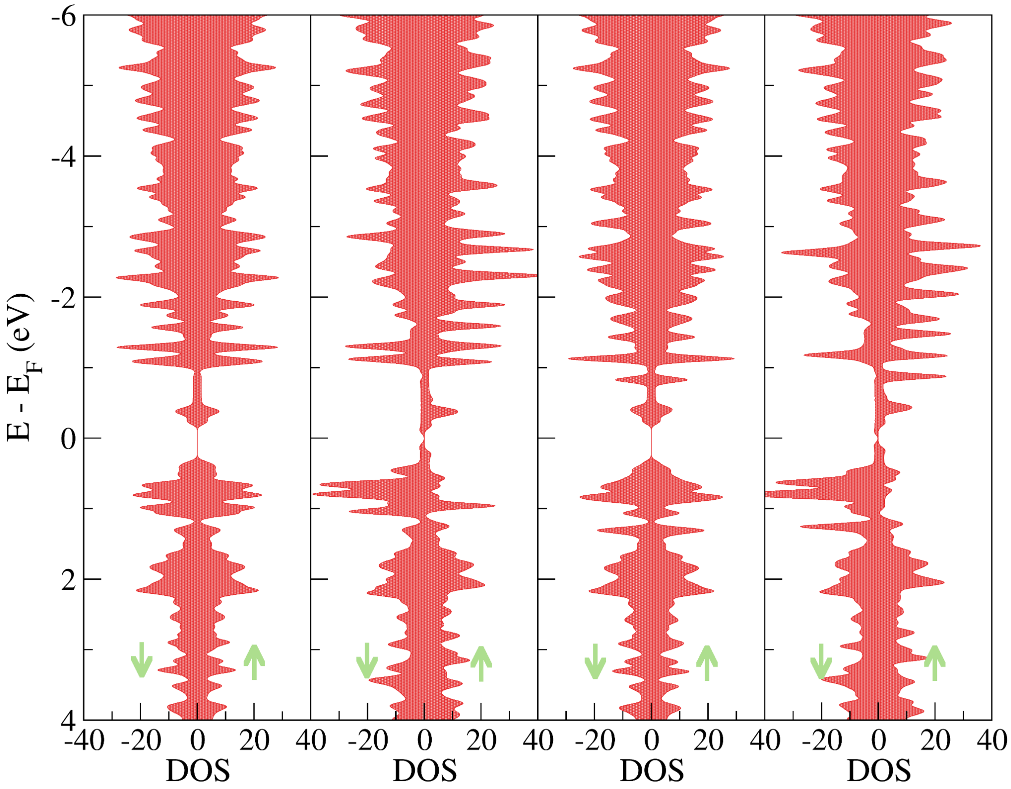

Figure S12. Total density of states (DOS) of selected **S1** cases. These states correspond to the same ones picked for the spin density analysis (Fig. S6). They are ordered from left to right according to their stability. Positive and negative values correspond to α and β channels, respectively. The Fermi energy (E_F) is set to zero in all cases.

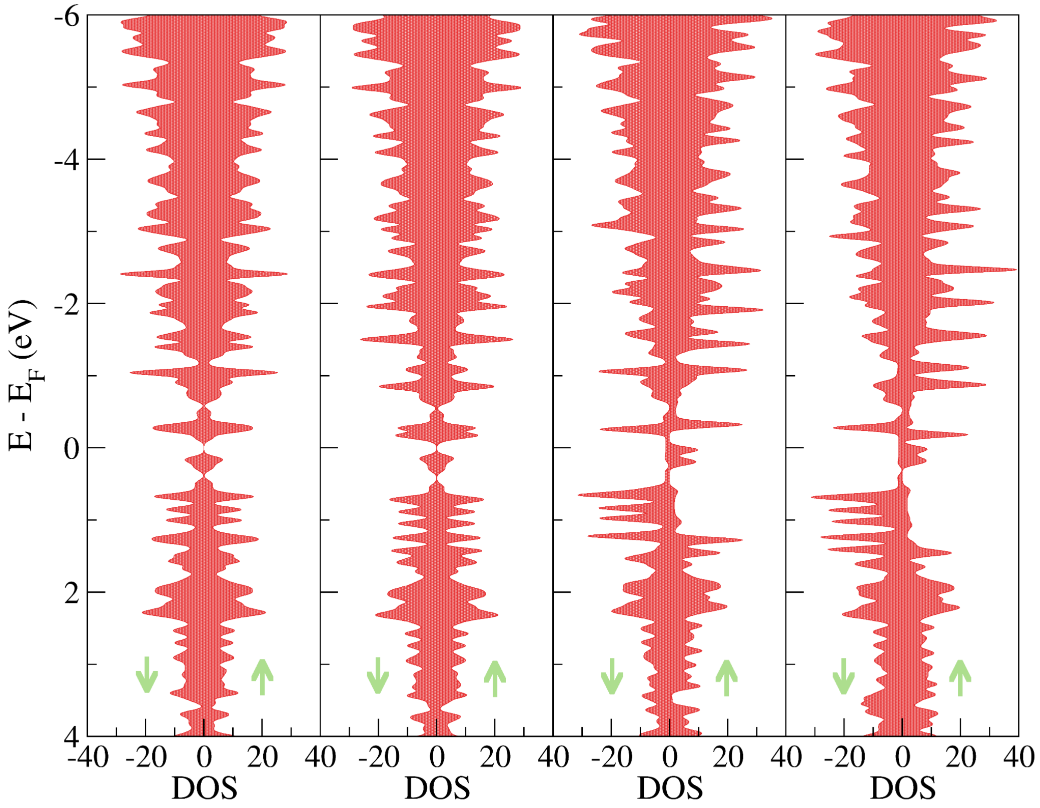

Figure S13. Total density of states (DOS) of selected **P1** cases. These states correspond to the same ones picked for the spin density analysis (Fig. S6). They are ordered from left to right according to their stability. Positive and negative values correspond to α and β channels, respectively. The Fermi energy (E_F) is set to zero in all cases.

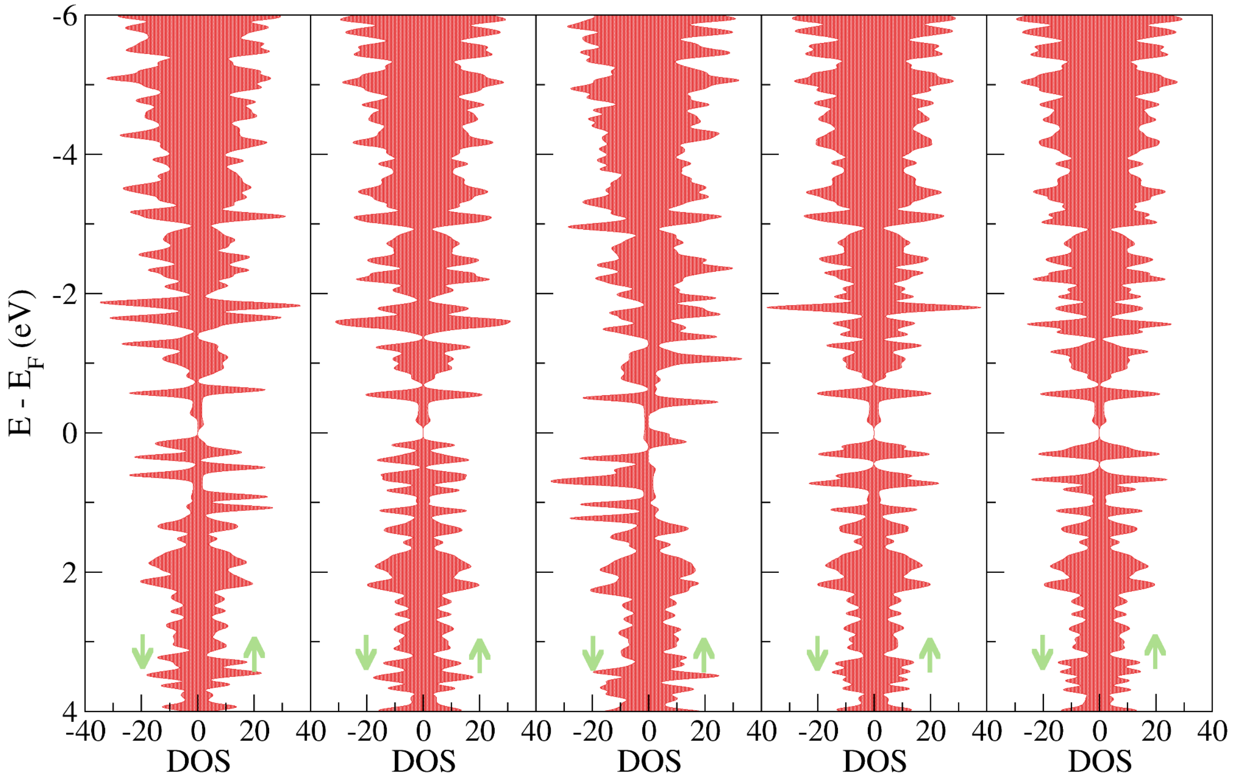

Figure S14. Total density of states (DOS) of selected **P2** cases. These states correspond to the same ones picked for the spin density analysis (Fig. S6). They are ordered from left to right according to their stability. Positive and negative values correspond to α and β channels, respectively. The Fermi energy (E_F) is set to zero in all cases.

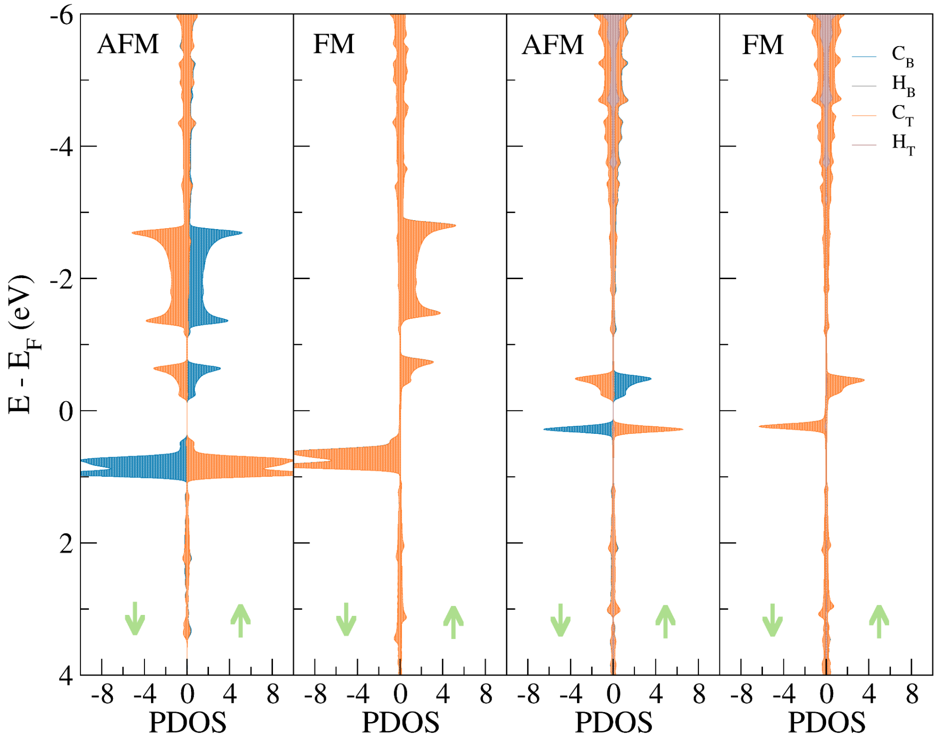

Figure S15. Projected density of states (PDOS) of undoped ZGNRs. The two leftmost PDOS correspond to unpassivated ZGNRs while the two rightmost ones correspond to passivated ZGNRs. AFM coupling is more stable in both cases. Positive and negative values correspond to α and β channels, respectively. The Fermi energy (E_F) is set to zero in all cases.

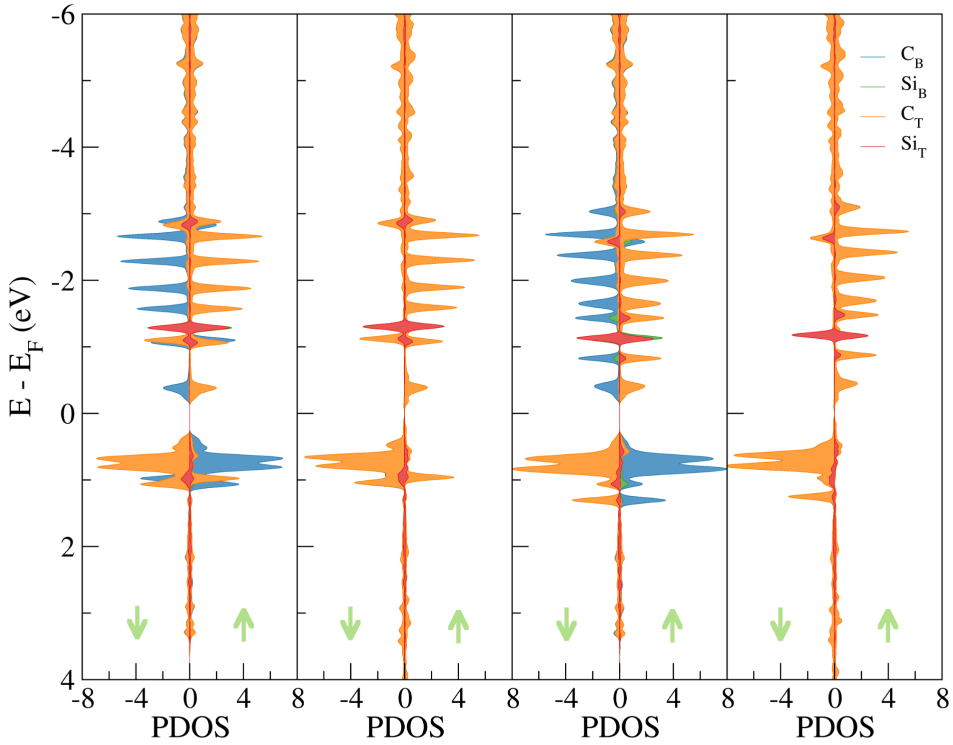

Figure S16. Projected density of states (PDOS) of selected **S1** cases. These states correspond to the same ones picked for the spin density analysis (Fig. S6). They are ordered from left to right according to their stability. Positive and negative values correspond to α and β channels, respectively. The Fermi energy (E_F) is set to zero in all cases.

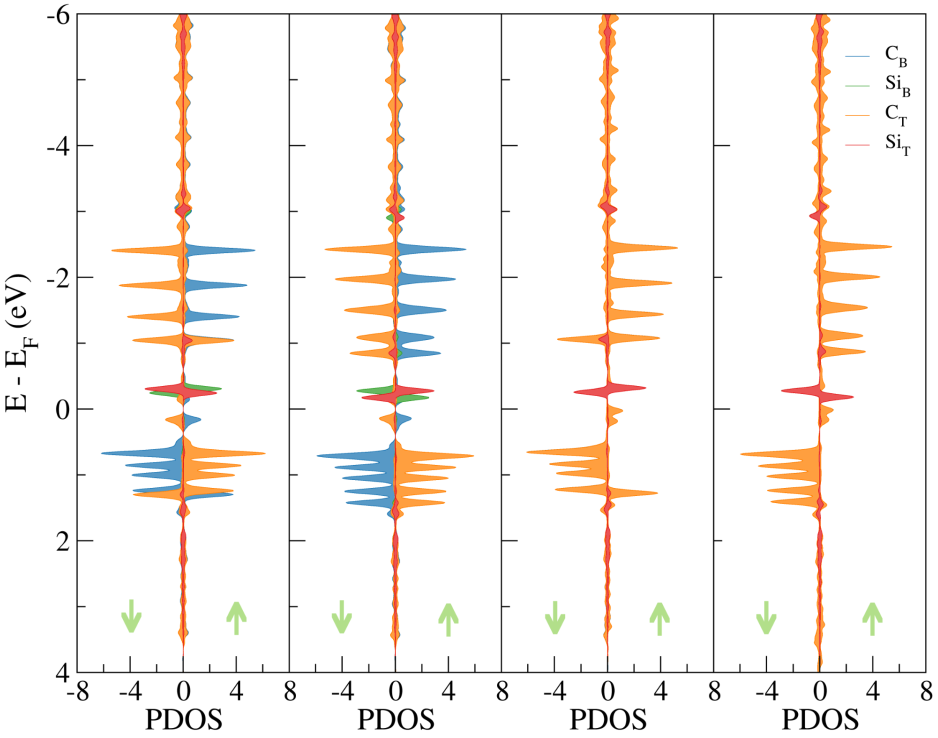

Figure S17. Projected density of states (PDOS) of selected **P1** cases. These states correspond to the same ones picked for the spin density analysis (Fig. S6). They are ordered from left to right according to their stability. Positive and negative values correspond to α and β channels, respectively. The Fermi energy (E_F) is set to zero in all cases.

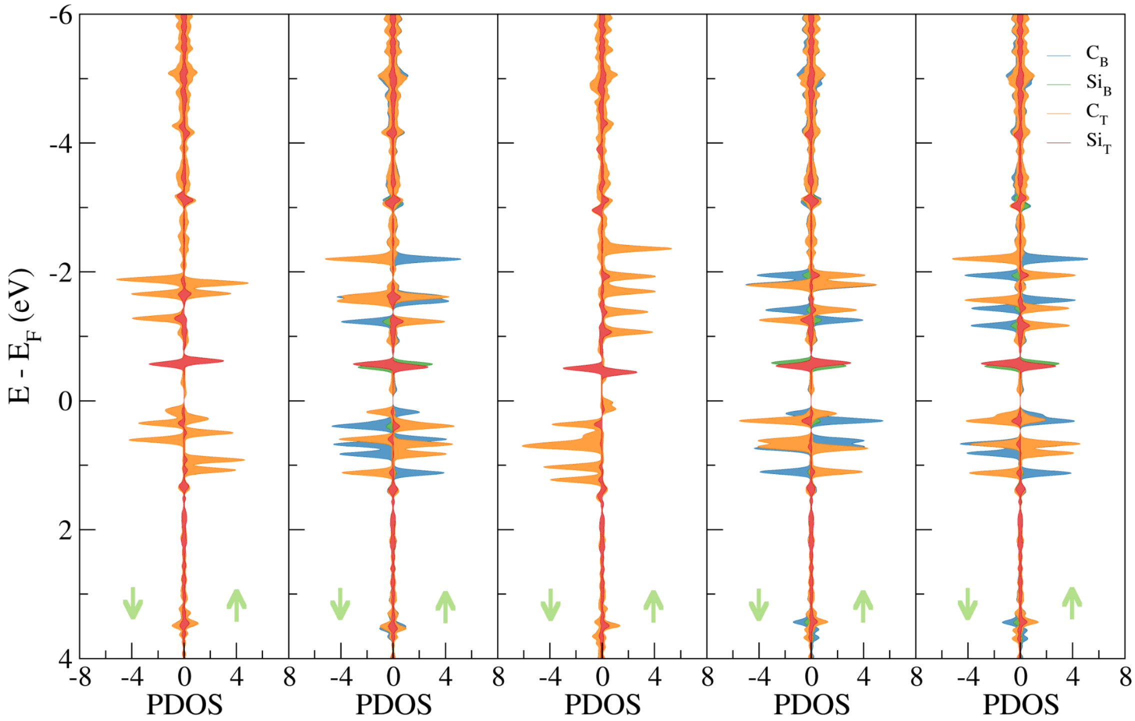

Figure S18. Projected density of states (PDOS) of selected **P2** cases. These states correspond to the same ones picked for the spin density analysis (Fig. S6). They are ordered from left to right according to their stability. Positive and negative values correspond to α and β channels, respectively. The Fermi energy (E_F) is set to zero in all cases.
